# Pathogenic effect of interleukin-17A in induction of Sjögren's syndrome-like disease using adenovirus-mediated gene transfer

**DOI:** 10.1186/ar3207

**Published:** 2010-12-23

**Authors:** Cuong Q Nguyen, Hongen Yin, Byung Ha Lee, Wendy C Carcamo, John A Chiorini, Ammon B Peck

**Affiliations:** 1 Eli and Edythe L. Broad Institute, 7 Cambridge Center, Cambridge, MA 02142, USA; 2 Department of Chemical Engineering, Massachusetts Institute of Technology, 77 Massachusetts Ave, E25-545, Cambridge MA 02139, USA; 3 Department of Oral Biology, University of Florida College of Dentistry, 1600 SW Archer Rd, Gainesville, FL 32610, USA; 4 Center for Orphan Autoimmune Disorders, University of Florida College of Dentistry, 1600 SW Archer Rd, Gainesville, FL 32610, USA; 5 National Institute of Dental and Craniofacial Research, NIH, 10 Center Drive MSC 1190, Bethesda, MD 20892, USA; 6 Department of Pathology, Immunology & Laboratory Medicine, University of Florida College of Medicine, 1600 SW Archer Rd, Gainesville, FL 32610, USA

## Abstract

**Introduction:**

Sjögren's syndrome (SS) involves a chronic, progressive inflammation primarily of the salivary and lacrimal glands leading to decreased levels of saliva and tears resulting in dry mouth and dry eye diseases. Seminal findings regarding T_H_17 cell populations that secrete predominantly interleukin (IL)-17A have been shown to play an important role in an increasing number of autoimmune diseases, including SS. In the present study, we investigated the function of IL-17A on the development and onset of SS.

**Methods:**

Adenovirus serotype 5 (Ad5) vectors expressing either IL-17A or LacZ were infused via retrograde cannulation into the salivary glands of C57BL/6J mice between 6 and 8 weeks of age or between 15 and 17 weeks of age. The mice were characterized for SS phenotypes.

**Results:**

Disease profiling indicated that SS-non-susceptible C57BL/6J mice whose salivary glands received the Ad5-IL17A vector developed a SS-like disease profile, including the appearance of lymphocytic foci, increased cytokine levels, changes in antinuclear antibody profiles, and temporal loss of saliva flow.

**Conclusions:**

Induction of SS pathology by IL-17A in SS-non-susceptible mice strongly suggests that IL-17A is an important inflammatory cytokine in salivary gland dysfunction. Thus, localized anti-IL17 therapy may be effective in preventing glandular dysfunction.

## Introduction

Sjögren's syndrome (SS) is a chronic, systemic autoimmune disease characterized most notably by development of dry eyes and dry mouth manifestations, indicative of secretory dysfunction of the lacrimal and salivary glands [1-3]. Although the etiology of SS remains unknown, intensive studies of an ever-expanding number of animal models is beginning to unravel the genetic, molecular and immunological basis for this disease [1]. Previous studies have implicated critical roles for both interferon-γ (IFN-γ) and interleukin (IL)-4 in the development and onset of SS-like disease in NOD/LtJ and C57BL/6.NOD-*Aec1Aec2 *mice [4,5], strongly suggesting involvement of T_H_1 and T_H_2 cell populations, respectively. While IFN-γ regulates cell-mediated immunity through activation of macrophages, NK cells and CD8^+ ^T cells, this cytokine appears to predispose these SS-susceptible mice by retarding salivary gland organogenesis, especially proliferation of acinar tissue [5]. This delay in acinar cell maturation has been postulated to prevent expression of cellular antigens at the critical time of self-tolerance, resulting in inefficient clonal deletion of acinar tissue-reactive T cells. In contrast to the role of IFN-γ both prior to and during development of SS, IL-4 appears to be essential during development of adaptive immunity and subsequent onset of glandular dysfunction. Specifically, IL-4 was shown to be necessary for proper isotypic switching, regulating B lymphocyte synthesis of pathogenic IgG1 anti-muscarinic acetylcholine type III receptor (M3R) autoantibodies [6,7].

Although these earlier studies have implicated both T_H_1 and T_H_2 cell-associated functions in the development and onset of clinical SS, recent identification of the CD4^+ ^T_H_17 memory cells within the lymphocytic focus (LF) of lacrimal and salivary glands of SS^s ^C57BL/6.NOD-*Aec1Aec2 *mice, as well as minor salivary glands of human SS patients, greatly expands the potential complexity in deciphering the autoimmune response underlying SS [8,9]. The T_H_17 cell population, while clearly a subset of CD4^+ ^memory effector T cells, appears to be distinct from, and unrelated to, either the T_H_1 or T_H_2 cell lineages [10-14]. T_H_17 effector cells secrete at least one of the six cytokines belonging to the IL-17 family, that is, IL-17A, IL-17B, IL-17C, IL-17D, IL-25 and/or IL-17F; however, IL-17A, the signature cytokine, has received the greatest attention in studies of autoimmune diseases [15]. The IL-17 cytokines are potent pro-inflammatory molecules, actively involved in tissue inflammation via induction of pro-inflammatory cytokine and chemokine expressions [16]. In addition, IL-17 is involved in the mobilization, maturation and migration of neutrophils via the release of IL-8 at the site of injury [17]. Interestingly, IL-17A is known to regulate Foxp3+ T_Reg _cells and vice versa [18].

While T_H_17 cells have been implicated in several autoimmune diseases (for example, Crohn's disease [19,20], experimental autoimmune encephalomyelitis (EAE) [21], collagen-induced arthritis (CIA) [21], SS [8] and others [2,3]), this characteristic may require signaling from T_H_1 cells already present in the lesion [3]. In any event, recent observational studies in SS patients and animal models of primary SS have identified the presence of IL-17A and its activating cytokine IL-23 in the lymphocytic infiltrates of the exocrine glands, as well as higher levels of circulating IL-17A in both sera and saliva [8], raising the question of the importance of IL-17 in SS. Thus, the goals of the present study were to determine whether IL-17A can directly influence the pathology leading to the onset of SS-like disease by administrating exogenous IL-17A to the salivary glands at specific time points.

## Materials and methods

### Animals

SS non-susceptible C57BL/6J mice were bred and maintained under specific pathogen-free conditions. The animals were maintained on a 12-hr light-dark schedule and provided food and acidified water *ad libitum*. At times indicated in the text, mice were euthanized by cervical dislocation following deep anesthetization with isoflurane, after which organs were freshly explanted for analyses. Both the breeding and use of these animals for the present studies were approved by the University of Florida's IACUC and IBC. Salivary glands of mice were cannulated with mouse IL-17A-expressing Ad5-IL17A vector using retrograde injections at either 7 weeks (wks) of age (*n *= 11) or 16 wks of age (*n *= 8). In addition, mice at 6 wks (*n *= 4) and 15 wks (*n *= 4) were randomly selected and used as pre-treated or baseline analysis. Age- and sex-matched control C57BL/6J mice (*n *= 10 per age group) received the Ad5-LacZ control vector using the same protocols.

### Production of Ad5-LacZ and Ad5-IL17A vectors

The recombinant adenovirus vectors used in this study were generously provided by Dr. Jay K. Kolls (Children's Hospital of Pittsburgh, Pittsburgh, PA, USA). These vectors are based on the first generation adenovirus serotype 5 (Ad5) and shown to produce their appropriate and functional mouse IL- 17A and LacZ products [22-24]. To obtain sufficient viral vectors for the present studies, each recombinant vector was amplified in HEK293 cells, purified by two rounds of CsCl gradient centrifugation, then dialyzed against 100 mM Tris-HCl (pH 7.4), 10 mM MgCl_2 _and 10% (v/v) glycerol, as described elsewhere [25].

### Retrograde salivary gland cannulation of Ad5-LacZ or Ad5-IL17A vectors

Previous studies have demonstrated that retrograde salivary gland cannulation is an effective method to direct local gene expression in the salivary glands [26-28]. In brief, prior to cannulation, each mouse was anesthetized with a ketamine:xylazine mixture (100 mg/mL, 1 mL/kg body weight; Fort Dodge Animal Health, Fort Dodge, IA, USA) and xylazine (20 mg/mL, 0.7 mL/kg body weight; Phoenix Scientific, St. Joseph, MO, USA)) intramuscularly. Stretched PE-10 polyethylene tubes were inserted into each of the two openings of the salivary ducts. After securing the cannulas, the mouse received an intramuscular injection of atropine (1 mg/kg), followed 10 minutes later by a slow, steady injection of viral vector. Each salivary gland received 50 μl of vector solution containing 10^7 ^viral particles). The cannulas were removed five minutes later to ensure successful cannulation.

### Measurement of saliva flow

To measure stimulated saliva flow, individual non-anesthetized mice were weighed and given an intraperitoneal injection of 100 μl of phosphate-buffered saline (PBS) containing isoproterenol (0.02 mg/ml) and pilocarpine (0.05 mg/ml). Saliva was collected for 10 minutes from the oral cavity of individual mice using a micropipette starting 1 minute after injection of the secretagogue. The volume of each saliva sample was measured. Prior to vector cannulation and again at each time-point designated in the text, saliva and sera were collected from each mouse. Samples were stored at -80°C until analyzed.

### Determination of cytokines levels

Measurements of IL-6 and IL-17A cytokine levels in sera samples were performed by an independent contractor (Millipore, Billerica, MA, USA) using Luminex^® ^platform.

### Intracellular cytokine staining and flow cytometric analysis

Spleens were freshly explanted, gently minced through stainless steel sieves, suspended in PBS and centrifuged (1,200 rpm for five minutes). Erythrocytes were lysed by seven-minute incubation in 0.84% NH4Cl. The resulting leukocyte suspensions were washed two times in PBS, counted and resuspended inculture media (RPMI 1640 medium, 10% FBS, 2 mM L-glutamine, 0.05 mM β-mercaptoethanol) at a density of 2 × 10^6 ^cells/ml. One million cells were pipetted to individual wells of a 24-well microtiter plate pre-coated with anti-CD3 (10 μg/ml) and anti-CD28 antibodies (2 μg/ml) for T cell activation. Cells were incubated for five hours with Leukocyte Activation Cocktail containing GolgiPlug (2 μl/ml). Collected cells were fixed and permeabilized using Cytofix/CytopermFixation/Permeabilization. Flow cytometric acquisition for intracellular staining was performed following staining with PE-Cy5-conjugated anti-mouse CD4, FITC-conjugated anti-IFN-γ and PE-conjugated anti-IL-17AA. The cells were counted on a FACSCalibur (BD, Franklin Lakes, NJ, USA) and analyzed by FCS Express (De Novo Software, Los Angeles, CA, USA).

### Histology

Following euthanasia, whole salivary glands containing submandibular, sublingual, and parotid glands were surgically removed from each mouse and placed in 10% phosphate-buffered formalin for 24 hrs. Fixed tissues were embedded in paraffin and sectioned at 5 μm thickness. Paraffin-embedded sections were de-paraffinized by immersing in xylene, followed by dehydrating in ethanol. The tissue sections were prepared and stained with hematoxylin and eosin (H&E) dye. Stained sections were observed under a microscope for glandular structure and leukocyte infiltration determination. A double-blinded procedure was used to enumerate leukocytic infiltrations (lymphocytic foci) in the histological sections of salivary glands. Lymphocytic foci (LF) were defined as aggregates of >50 leukocytes quantified per each histological section. Calculations were based on one histological section per mouse.

### Immunofluorescent staining for CD3+T cells and B220+B cells

Histological sections of salivary glands were incubated with rat anti-mouse B220 (BD Pharmingen, San Jose, CA, USA) and goat anti-mouse CD3 (Santa Cruz Biotechnology, Santa Cruz, CA, USA), followed by incubation with Texas Red-conjugated rabbit anti-rat IgG (Biomeda, Foster City, CA, USA) and FITC-conjugated rabbit anti-goat IgG (Sigma-Aldrich, St. Louis, MO, USA). The slides were mounted with DAPI-mounting medium (Vector Laboratories, Burlingame, CA, USA). Sections were observed at 200X magnification using a Zeiss Axiovert 200 M microscope.and images were obtained with AxioVs40 software (Ver. 4.7.1.0, Zeiss) (Carl Zeiss, Thornwood, NY, USA). Enumeration of B, T cells and total number of nuclei in the LF were performed using Mayachitra imago software (Mayachitra, Inc, Santa Barbara, CA, USA).

### Immunohistochemical staining for IL17A in salivary glands

Immunohistochemical staining for IL17A were carried out as previously described [8]. In brief, paraffin-embedded salivary glands were deparaffinized by immersion in xylene, followed by antigen retrieval with 10 mM citrate buffer, pH 6.0. Tissue sections were incubated overnight at 4°C with anti-IL-17A antibody (Santa Cruz Biotechnology). Isotype controls were done with rabbit IgG. The slides were incubated with biotinylated goat anti-rabbit IgG followed by horseradish peroxidase-conjugated strepavidin incubation using the Vectastain ABC kit. The staining was developed by using diaminobenzidine substrate (Vector Laboratories), and counterstaining was performed with hematoxylin. Sections were observed at 200X magnification using a Zeiss Axiovert 200 M microscope. And images were obtained with AxioVs40 software (Ver. 4.7.1.0, Zeiss) (Carl Zeiss). Enumeration of IL17A-positive cells was performed on the entire histological sections of the whole salivary glands using Mayachitra imago software (Mayachitra, Inc.), although lymphocytic infiltrations are normally seen only in the submandibular glands.

### Detection of antinuclear antibodies (ANA) in the sera

ANA in the sera of mice were detected using HEp-2 ANA kit (INOVA Diagnostics, Inc., San Diego, CA, USA). All procedures were performed per manufacturer's instructions. In brief, HEp-2 fixed substrate slides were overlaid with appropriate mouse sera diluted 1:40, 1:80 and 1:160. Slides were incubated for one hour at room temperature in a humidified chamber. After three washes for five minutes with PBS, the substrate slides were covered with Alexa 488-conjugated goat anti-mouse IgG (H/L) (Invitrogen Inc, Carlsbad, CA, USA) diluted 1:100 for 45 minutes at room temperature. After three washes, fluorescence was detected by fluorescence microscopy at 200X magnification using a Zeiss Axiovert 200 M microscope and all images were obtained with AxioVs40 software with constant exposure of 0.3 seconds (Carl Zeiss). Negative controls are secondary antibody only and positive controls are standard serum with nuclear speckled pattern provided with the kits. Data presented in the results are from slides using 1:40 dilutions of sera from each experimental group.

### Statistical analyses

Statistical evaluations were determined by using the Mann-Whitney U test generated by the GraphPad InStat software (GraphPad Software, La Jolla, CA, USA). The two-tailed *P-*value < 0.05 was considered significant.

## Results

### Induction of IL-17A and IL-6 cytokine levels in sera following transduction with Ad5-IL17A vector

Adenoviral vectors have been reported to show peak gene expressions around Day 5 post-infusion and then persist for approximately two weeks [29]. In the current study, immunohistochemical staining for the presence of LacZ protein in the infused salivary glands demonstrated that optimal transduction efficiency was approximately 26 ± 5% at two weeks post-infusion which decreased to 15 ± 3% by nine weeks post-infusion. The cells within the salivary glands positive for LacZ expression were predominantly ductal cells, as expected, and acinar cells (data not shown), indicating the virus was capable of passing through the ducts.

**Figure 1:**
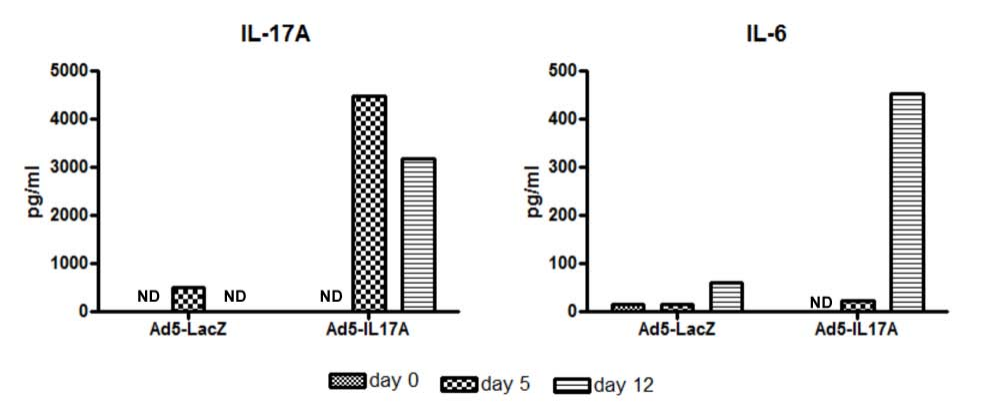
**Rapid changes in IL-17A and IL-6 serum cytokine concentrations in C57BL/6J mice following vector cannulations**. Sera were prepared from blood collected from individual five-week old mice (*n *= 4) randomly chosen one week prior to vector treatment (Day 0 on the graph). Mice were allowed to acclimate for seven days, followed by vector instillation of each salivary gland with 50 μl of vector solution containing 10^7 ^viral particles of either Ad5-LacZ or Ad5-IL17A vector. Sera were again prepared from blood collected from individual mice (*n *= 11) at Day 5 and Day 12 post-treatment. Concentrations of cytokines were determined using the Luminex platform. To ensure sufficient quantities for testing, the sera of three individual mice of each experimental group were pooled. ND, not detected indicates levels below threshold detection.

To determine if transduction of salivary glands with IL-17A alters the serum cytokine profiles, serum preparations were assessed for temporal changes in pro-inflammatory cytokine levels. Sera of treated mice were collected at Days 5 and 12 post-treatment to determine the efficacy of the IL-17A expressing viral vectors to affect cytokine secretions. As shown in Figure 1, C57BL/6J mice treated with the Ad5-IL17A vector at 10^7 ^viral particles per salivary gland exhibited a marked increase in the levels of serum IL-17A compared to baseline levels or with C57BL/6J mice receiving the control Ad5-LacZ vector at 10^7 ^viral particles per salivary gland, demonstrating the efficacy of this viral vector to produce IL-17A. In addition, Ad5-IL17A-treated C57BL/6J mice also secreted elevated amounts of the IL-17A-related cytokine IL-6 following cannulation. Thus, the vectors gain access into the glands and apparently secrete IL-17A in quantities that elevate systemic levels.

### Increased numbers of IL-17A-producing CD4+ T cells in the spleens of Ad5-IL17A transduced mice

As mentioned previously, salivary glands were cannulated with Ad5-IL17A vector at either 7 wks or 16 wks of age. The time points chosen are based on extensive studies of the development and onset of disease in our C57BL/6.NOD-*Aec1Aec2 *mouse model of SS [1-3,30,31]. The two time points selected represent the innate and adaptive immune response phases, respectively, in the disease model, thus they were chosen to mimic these changes in the parental C57BL/6 mouse. Microarray analyses examined the temporal differential gene expression of salivary and lacrimal glands of C57BL/6 mice revealed gradual change in pathophysiological related genes from 16 to 20 wks of age, concomitantly, leukocyte infiltration in the exocrine glands is often observed at these ages [32,33]. Thus, it is important to examine the role of IL17A in the development of SS prior and post to any pathophysiological changes.

**Figure 2:**
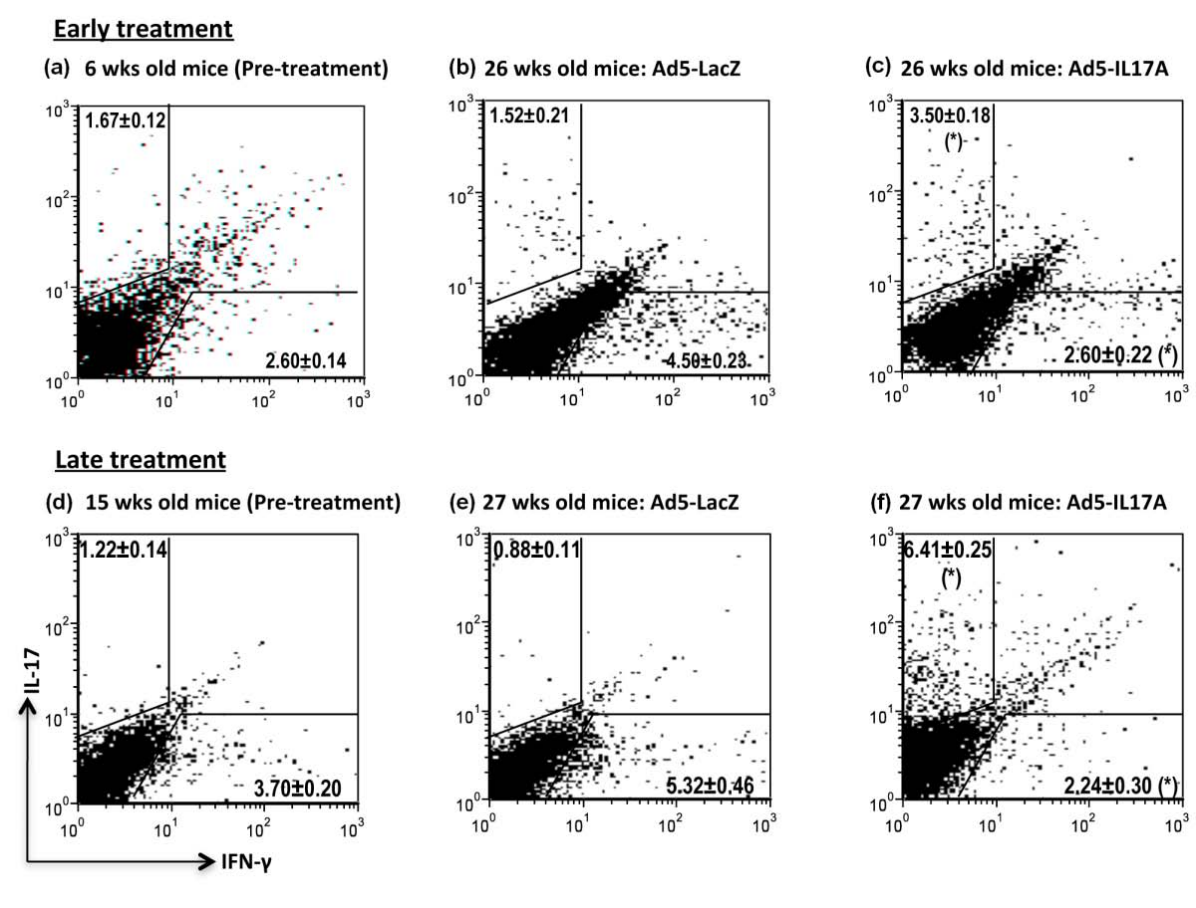
**Intracellular staining for IL-17A and IFN-γ secreting CD4**^**+**^**T cells in spleens of Ad5-IL17A-treated mice**. Splenic leukocytes prepared from C57BL/6J mice (*n *= 4) at 6 wks of age (one wk prior to vector treatment) and 26 wks old (19 wks post vector treatment), considered early treatment **(a-c)**, or splenic leukocytes prepared from C57BL/6J mice (*n *= 4) at 15 wks of age (1 wk prior to vector treatment) and 27 wks old (11 wks post vector treatment), considered late treatment **(d-f) **were examined for the presence of intracellular IL-17A and IFN-γ gated on CD4^+^T cells following a 5-hr *in-vitro *activation with anti-CD3ε and anti-CD28 in Leukocyte Activation Cocktail containing GolgiPlug. Flow cytometric acquisition was performed by staining with PE-Cy5-conjugated rat anti-CD4, FITC-conjugated rat anti-IFNγ and/or PE-conjugated rat anti-IL-17A. Data were analyzed by FCS Express. Flow cytometric images shown are from one representative analysis of two independent experiments that examined two different mice within each experiment. Data presented as mean ± SEM for *n *= 4 per group and statistical analyses were performed comparing the means of the Ad-LacZ and Ad-IL17A treated groups at 26 wks and 27 wks of early and late treatment, respectively. (*) indicates *P *< 0.5 using the Mann-Whitney U test.

Mice treated with Ad5-IL17A or Ad5-LacZ at either 7 wks or 16 wks of age were euthanized at 26 and 27 wks of age, that is, 19 wks and 11 wks post-treatment, respectively. Splenocytes were isolated from individual mice and examined for the number of IFN-γ and IL-17A secreting CD4+T cells. Representative data, presented in Figure 2b, c, revealed that the number of IL-17A secreting CD4+T cells in the spleens of mice receiving the Ad5-IL17A vector at seven weeks of age was approximately two-fold higher than mice receiving the control Ad5-LacZ vector, while the number of IFN-γ secreting CD4+T cells was approximately half at time of analysis. Similarly, the number of IL-17A secreting CD4+T cells in the spleens of mice receiving the Ad5-IL17A vector at 16 wks of age was approximately seven-fold higher than mice receiving the control Ad5-LacZ vector, while the number of IFN-γ secreting CD4+T cells was less than 50% at time of analysis (Figure 2e, f). Results of a similar analysis with untreated mice performed one week prior to vector cannulations are presented in Figures 2a, d. These data suggest that even though the Ad5 vector is considered locally restricted, the effect in C57BL/6 J mice appeared systematic. More importantly, the systemic effects of IL17A in Ad5 appears to be correlated with the duration of gene expression after vector cannulation as evidenced by the two-fold increase in the levels of IL-17A secreting cells at 19 wks post-treatment in younger mice but a seven-fold increase at 11 wks post-treatment in the older group. However, one cannot rule out the possibility that different efficacies are achieved based on the status of disease development in different ages of mice.

### Induction of SS immune-pathology in C57BL/6 mice following treatment with Ad5-IL17A vector

**Figure 3:**
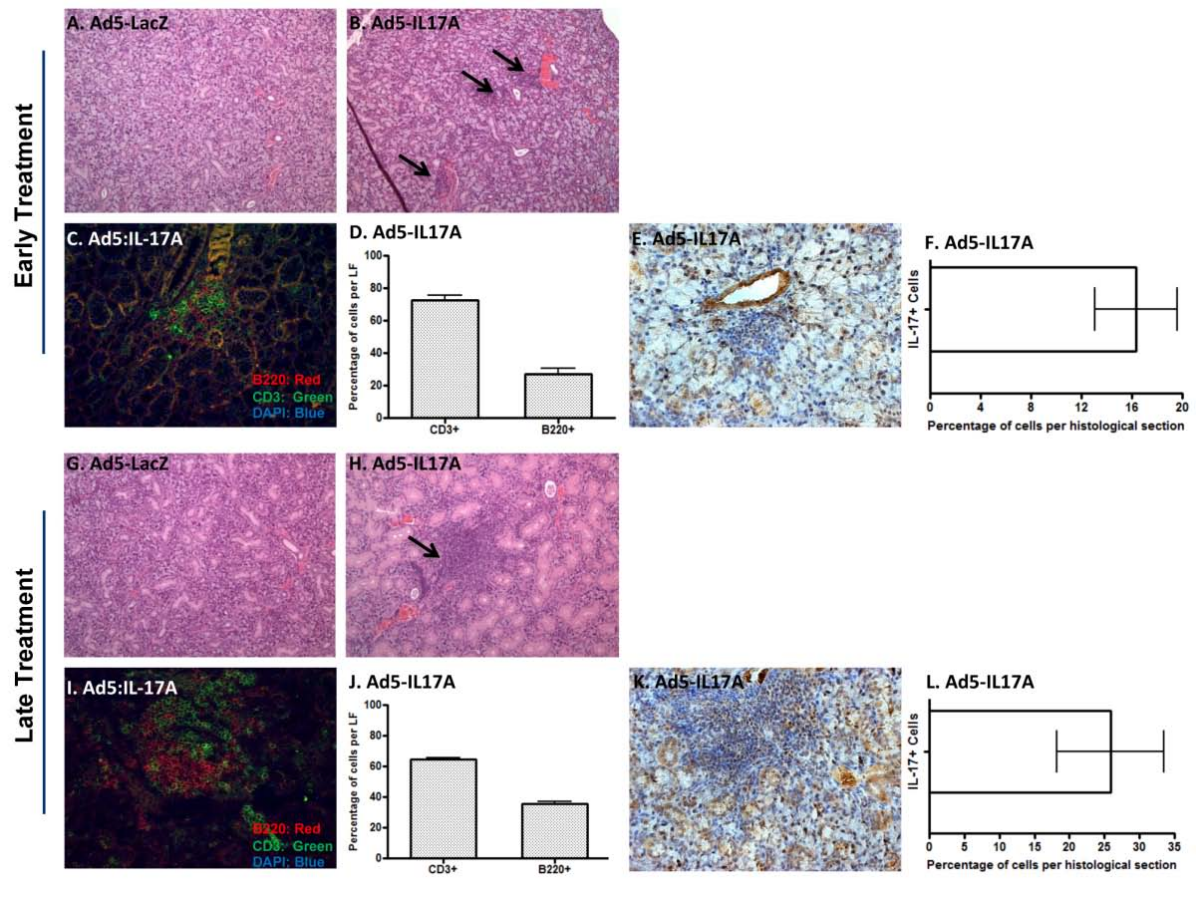
**Histological examination of salivary glands**. Salivary gland histology was examined at 19 wks post-vector infusions of mice treated at 7 wks of age (early treatment) or at 11 wks post-vector infusions of mice treated at 16 wks of age (late treatment). Panels show representative H&E staining of salivary gland tissue from mice receiving early treatment with Ad5-LacZ (*n *= 10) **(a)**, or Ad5-IL17A (*n *= 11) **(b)**; fluorescent staining and enumeration of B and T cells in Ad5-IL17A treated mice **(c and d) **and immunohistochemical staining and enumeration of IL-17A-positive cells in Ad5-IL17A treated mice **(e and f)**; H&E staining of salivary gland tissue from mice receiving late treatment with Ad5-LacZ (*n *= 10) **(g)**, or Ad5-IL17 (*n *= 8) **(h)**; and fluorescent staining and enumeration of B and T cells in Ad5-IL17A treated mice **(i and j) **and immunohistochemical staining and enumeration of IL-17A-positive cells in Ad5-IL17A treated mice **(k and l)**. Black arrows indicate representative lymphocytic infiltrate.

Lymphocyte infiltration of the salivary and/or lacrimal glands is a critical criterion for identification of the autoimmune phase of SS in both human and animal models. Although the number of LF present in the salivary and lacrimal glands does not often correlate directly with disease or its severity, SS patients and NOD-derived mouse strains exhibiting SS-like disease typically have lymphocytic infiltrates in their salivary glands. IL-17A appears to play a critical role in the development of LF and has recently been found to be present within LF in both SS patients and animal models [8]. Salivary glands of C57BL/6J mice following cannulation with Ad5-IL17A vector were examined for the presence of infiltrating leukocytes. Salivary glands retrieved from C57BL/6J mice treated with Ad5-LacZ vector at either 7 or 16 wks of age revealed that 10% (1 of 10) in each group had evidence of glandular infiltrations (Figure 3a, b, g, h; Table 1). This observation is consistent with the number of healthy, untreated C57BL/6J mice expected to have infiltration of the salivary glands [8]. In contrast, salivary glands from C57BL/6J mice treated with Ad5-IL17A vector at seven weeks of age showed infiltrations in 91% (10 of 11) with the mean LF per histological section numbering 4 ± 1.32, while salivary glands from C57BL/6J mice treated with Ad5-IL17A vector at 16 wks of age revealed infiltrations in 75% (6 of 8) with a mean LF number per histological section of 2 ± 0.83 (Table 1).

**Table 1:** Quantification of lymphocytic foci (LF) in salivary glands

	Ad5:LacZ			Ad5:IL17A		
		
	No LF	LF	Mean LF	No LF	LF	Mean LF
**Early**	9^a ^(90%)^b^	1 (10%)	1	1 (9%)	10 (91%)	4 ± 1.32^c^
**Late**	9 (90%)	1 (10%)	1	2 (25%)	6 (75%)	2 ± 0.83

^a^number of mice.

^b^percentage of mice.

^c^mean number of LF ± SEM per histological salivary gland section.

Ad5, Adenovirus serotype 5; IL, interleukin; LF, lymphocytic foci.

Besides the number of LF detected in the salivary glands of the experimental animals, immunofluorescent staining to detect B and T cells revealed further differences in the cellular composition of the infiltrations between mice administered Ad5-IL17A at an early or late stage. At time of euthanasia, C57BL/6J mice treated with Ad5-IL17A vector at 7 wks of age generally exhibited smaller foci containing fewer IL-17 positive cells compared to mice receiving the vector at 16 wks of age (Figure 3c-f, i-l). Consistent with previous observation, the smaller foci in mice treated at 7 wks of age may have resulted from the longer duration of time after cannulation (19 wks) reflecting the decreases in IL-17A serum levels and IL-17A- positive cell numbers. Detailed examination of IL-17A-positive cells revealed that a majority of IL-17A cells are present in the LF and ductal cells with smaller percentage of positive cells found in the epithelium and acinar cells. Nevertheless, these data support the concept that formation and maintenance of LF are due, in part, to the expression levels of IL17A in the salivary glands.

### Changes in ANA profiles following instillation of the Ad5-IL-17A vector

**Figure 4:**
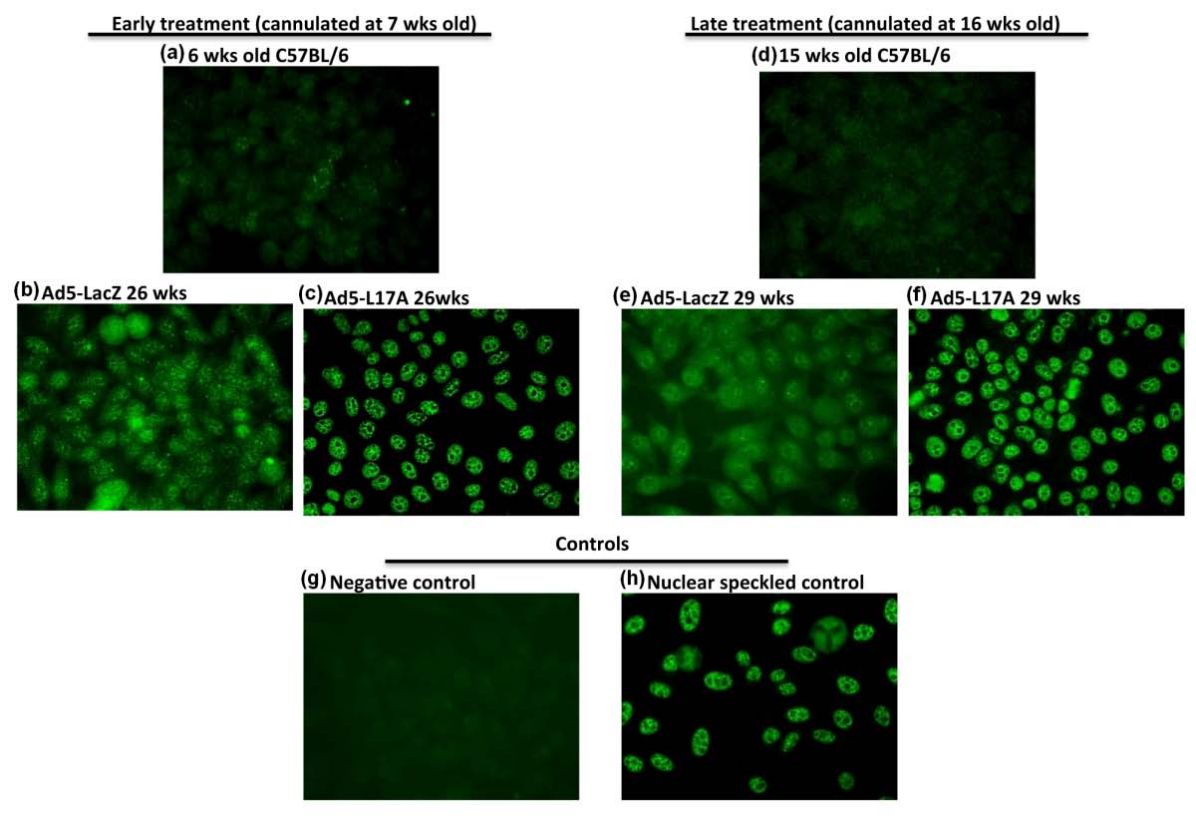
**Identification of the antinuclear antibodies in sera of C57BL/6J mice**. Representative patterns of cellular staining of HEp-2 cells by sera diluted at 1:40 prepared from sera taken from C57BL/6 mice cannulated with Ad5-LacZ or Ad5-IL17A vectors at 7 wks of age with pre-treated mice (baseline) at 6 wks of age (*n *= 4) **(a-c)**, and cannulated at 16 wks of age with Ad5-LacZ or Ad5-IL17A and pre-treated mice (baseline) at 15 wks of age (*n *= 4) **(d-f) **with negative control using secondary antibody only **(g) **and positive control with standard nuclear speckled serum **(h)**. Representative patterns were determined with *n *= 4 for two baselines and *n *= 7 for each time point presented in the figure. Fixed HEp-2 substrate slides were incubated with individual mouse sera diluted 1:40, 1:80 and 1:160 followed by development with FITC-conjugated goat anti-mouse IgG. Fluorescent patterns were detected by fluorescence microscopy at 400X magnification.

With the appearance of B and T lymphocytes within the salivary glands of Ad5-IL17A treated C57BL/6 mice, plus the significant changes within their splenic T_H_17 and T_H_1 cell populations, the presence of circulating autoantibodies, specifically ANA, detectable by staining of HEp-2 cells was examined. To identify the presence of ANA, the sera prepared from blood samples collected from each C57BL/6J mouse both pre- and post-cannulation were tested for reactivity on HEp-2 cells. As presented in Figure 4a, the sera collected from C57BL/6J mice at six weeks of age or one week prior to vector treatment showed a general weakly diffused cytoplasmic and nuclear background staining of the individual target cells. However, sera collected 19 wks post-treatment from mice treated with Ad5-IL17A vector at 7 wks of age showed no cytoplasmic staining with course speckled staining and negative nucleoli, while Ad5-LacZ treated mice exhibited diffused cytoplasmic staining, weak but fine speckled nucleoplasmic staining with negative nucleoli (Figures 4b, c). Similar results were seen in C7BL/6J mice whose salivary glands were transduced with Ad5-IL17A vector at 16 wks of age in which the pattern was pronounced course speckled staining with no cytoplasmic staining and negative nucleoli at 29 wks of age, or 11 wks post-treatment (Figures 4d-f). Considering the functions of IL-17A, it is interesting to see a gradual and subtle change in ANA profile from diffused cytoplasmic/nuclear pattern to a distinct course nuclear speckled pattern, suggesting influence of IL-17A on the B cells repertoire.

### Induction of salivary gland dysfunction in C57BL/6J mice following cannulation with Ad5-IL17A vector

**Figure 5:**
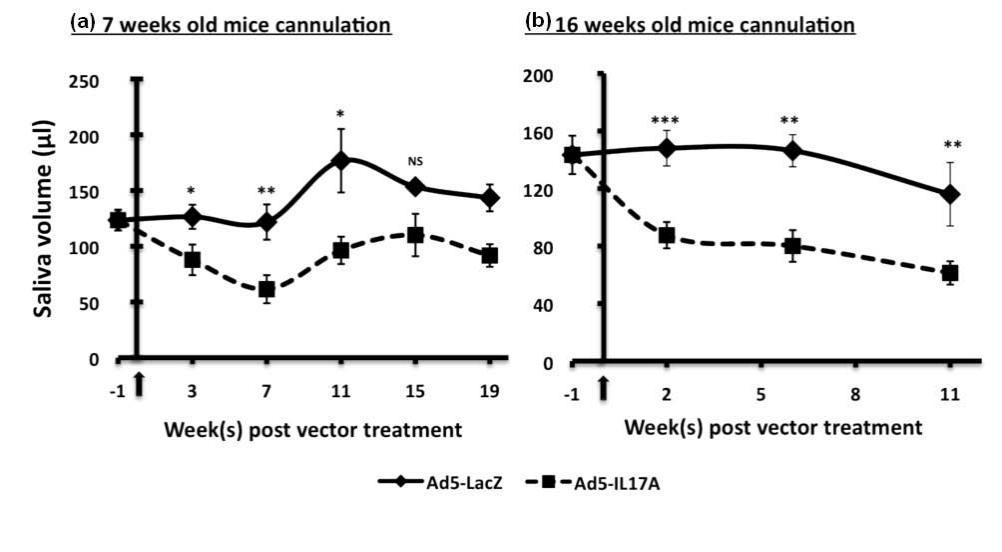
**Stimulated saliva flow in treated C57BL/6J mice**. One week prior to salivary gland cannulations with either Ad5-LacZ or Ad5-IL17A vector, stimulated saliva volumes were determined for individual mice within each of the four experimental groups: early treatment with Ad5-LacZ (*n *= 10) or Ad5-IL17A (*n *= 11) at 7 wks of age **(a) **or late treatment with Ad5-LacZ (*n *= 10) or Ad5-IL17A (*n *= 8) at 16 wks of age **(b)**. Saliva was collected every three to five weeks post-treatment until the mice were euthanized. Statistical analysis was used to determine the significance between the Ad5-LacZ and Ad5-IL17A treated mice at each time point. (NS: not significant, *P *= *< 0.05, *P *= **< 0.01, *P *= ***< 0.001). Arrows indicate the initial time point of vector cannulation.

To determine if the expression of exogenous IL-17A can induce salivary gland dysfunction, saliva volumes for each mouse were measured at one week prior to treatment, then at three- to five-week intervals post-cannulation. C57BL/6J mice that received control Ad5-LacZ vector at seven weeks of age exhibited stable stimulated saliva volumes at seven weeks post treatment with a statistically non-significant increase in saliva volumes at 11 weeks post treatment. Nevertheless, C57BL/6J mice whose salivary glands were cannulated at seven weeks of age with Ad5-IL17A exhibited a significant and relatively rapid decrease in stimulated saliva volumes that was most pronounced at seven weeks post treatment, and this observation is seen even if the saliva volumes are converted to saliva flow rates based on weights of the mice. After seven weeks post treatment, these mice showed a slight recovery (Figure 5a). Similar results were observed with C57BL/6J mice cannulated at 16 wks of age with Ad5-LacZ and Ad5-IL17A vectors; however, no saliva volume recovery was observed at time of euthanization (that is, 11 wks post-treatment) (Figure 5b). Whether a reversal of this inhibition would occur in these older animals will require further studies. Thus, saliva secretions of mice receiving the Ad5-IL17A vector were significantly decreased one to two months post-treatment when compared to secretions of mice receiving the Ad5-LacZ vector.

## Discussion

The T_H_17-derived IL-17A cytokine is a potent inflammatory cytokine that has been implicated in a growing list of autoimmune diseases, for example, multiple sclerosis, Crohn's disease, rheumatoid arthritis, psoriasis, systemic lupus erythematosus, and SS, as well as autoimmunity in animal models [3]. As the T_H_17/IL-17A system is considered to be an important factor in innate immunity that, in turn, regulates development of the adaptive immune response, it is not surprising that environmental microflora trigger IL-17A responses [34]. The consequence of T_H_17/IL-17A activation includes, in addition to the production the IL-17A family of cytokines, the production of IL-21, IL-22, chemokines (MIP-2, CXCL1, CXCL2, CXCL5), and matrix metalloproteases (MMP3 and MMP13) [16] all actively involved in tissue inflammation. Interaction of the IL-17A with its receptors evokes activation of IL-8, resulting in recruitment of neutrophils to the site of injury. However, the relationship between such early innate/inflammatory events mediated by the T_H_17/IL-17A system and the role T_H_17 cells play in subsequent autoimmunity remains unknown, especially in light of the multiple functions now associated with the T_H_17 cell populations. Thus, in the present study, we have attempted to elucidate the importance of the cytokine IL-17A *per se *in the development of SS and whether its function may be dependent on when it is expressed.

Results in which SS-non-susceptible C57BL/6J mice were cannulated with the Ad5-IL17A vector revealed that increased IL-17A expression could induce several pathological features of SS, irrespective of whether the mice received the vector at 7 or 16 wks of age, two time points corresponding to innate and adaptive immune responses in SS-susceptible C57BL/6.NOD-*Aec1Aec2 *mice. This was noted by decreases in saliva production compared to control vector, elevated production of specific pro-inflammatory cytokines detected in sera, changes in the weak cytoplasmic/nuclear ANA patterns to nuclear specked staining on HEp2 cells and increased numbers of LF and IL17A positive cells present in the salivary glands at time of euthanasia. Interestingly, mice received Ad5-IL17A at 7 wks of age showed a slight recovery of saliva secretion at 7 wks of treatment in contrast to mice received Ad5-IL17A at 16 wks of age. This observation might be supported by the differential immunological or biological response of mice at different ages and the effect of Ad5-IL17A exerted on the mice.

Previous studies have indicated that genes placed within Ad5 vectors are generally expressed transiently and locally restricted (that is, 7 to 14 days) [29]. The present study demonstrates that a rapid and significant increase in the levels of plasma IL-17A was affected at 12 days post-cannulation by the Ad5-IL17A transgene vector. Interestingly, this systemic increase in IL17 cytokine levels correlated with significant increases in splenic IL-17A secreting CD4+T cells that persisted at least 19 wks for mice treated at 7 wks of age and 11 wks for mice treated at 16 wks of age. These observations indicated that the Ad5 vector effect was longer than anticipated. Whether this effect might be due to an indirect secondary effect of the Ad5-IL17 vector is unknown. In addition, the systemic increase in IL17A production by local treatment of Ad5-IL17A presented in this study is consistent with previous studies by Bruce Baum's laboratory [35-38]. Adesanya *et al*. [39] has demonstrated that acinar cells can be punctured by retrograde salivary gland cannulation at a certain vector dosage. The injured acinar cells, which have compromised mucosal barrier integrity, allow for leakage of the vector systemically. Further studies by Kagami *et al*. [37] and He *et al*. [40] provided evidence that ductal cannulation of salivary glands can also have systemic effects due to the secretory nature of the salivary glands which are well endowed with protein synthesis organelles and secretory machinery.

Nevertheless, these observations are consistent with the concept that SS develops along specific biological processes in a sequential fashion and interference with this process alters development of disease [1-3]. Therefore, this study clearly indicates the pathogenic nature of IL-17A in inducing SS-like phenotypes when cannulated in the salivary glands.

Previous data have shown that lymphocytic infiltrates in the salivary glands secreting IL-17A and its related cytokines are more important in local glandular destruction. Staining salivary glands for IL-17A revealed that C57BL/6J mice receiving Ad5-IL17A vector not only expressed significant levels of IL-17A, but that IL-17A levels correlated with recruitment of inflammatory cells, specifically B and T cells, to the glands. This observation is important in light of the recent study suggesting IL-17A is a critical factor in the adaptive immune response by inducing the formation of germinal centers for the production of autoreactive antibodies [24]. Autoantibodies represent a major component in the onset of SS, thus the changes in the ANA profiles observed with sera of C57BL/6J mice cannulated with the Ad5-IL17A vector indicate that IL-17A affects even the B cell compartment in SS-non-susceptible mice. The presence of LF and loss of saliva secretion raises an interesting question about the possible role of IL-17A in B cell activation. As BAFF is capable of inducing T_H_17 cell differentiation in addition to regulating B cell activation [41], the possible role of BAFF and IL17A in this phenomenon needs to be better defined in SS pathogenesis.

## Conclusions

The capability of IL-17A to induce features of SS in SS-non-susceptible mice demonstrates the major role this cytokine plays in the development, and possible onset, of the autoimmune process. How this one cytokine affects the various features of autoimmunity, and at what level or time point, will require additional studies. More importantly, the study demonstrates that IL-17A might be a potential therapeutic target for SS.

## Abbreviations

Ad5: adenovirus serotype 5; ANA: antinuclear antibodies; BAFF: B cell activating factor; CIA: collagen-induced arthritis; CXCL1: chemokine (C-X-C motif) ligand; EAE: experimental autoimmune encephalomyelitis; IFN-γ: interferon-γ; IL: interleukin; LF: lymphocytic focus; MIP-2: macrophage inflammatory protein-2; MMP: matrix metalloproteases; SS: Sjögren's syndrome.

## Competing interests

The authors declare that they have no competing interests.

## Authors' contributions

JAC produced and determined the titers of the Ad5-LacZ and Ad5-IL17A viral vectors. HY and BL performed retrograde ductal cannulations/instillations of the vectors into the salivary glands. CQN designed the study, performed saliva flow, flow cytometry, histology and statistical analyses, and prepared the manuscript. WC carried out the ANA staining. ABP assisted in the manuscript preparation. All authors read and approved the final manuscript.
